# Social Detectives

**Published:** 1892-01-09

**Authors:** 


### THE DOCTOR'S ARMCHAIR.

### SOCIAL DETECTIVES.

"WE make too much of faults." That is Carlyle's dictum; and it must be looked upon as a kind of confession. No man ever found more fault with the world or with human creatures than Carlyle. Literary men, poets, preachers, and statesmen who occupied very considerable eminences in the public estimation, were often disposed of by him with a mere snort, or a single contemptuous word. They were "futilities," "windbags," "poor creatures," "Dukes of Windlestraw," to say nothing of terms and labels still more offensive and opprobrious. But by the time that Carlyle had reached the age of forty-five he had begun to use better eyes than the jaundiced optics of Craigenputtock.

If there are beings in any neighbouring planet, who can see us and our doings from their outside post of observation, it is probable that nothing will surprise them more in our behaviour than the keen relish we have in the discovery and exposure of one another's faults. Well might Shakespeare say, "The evil that men do lives after them, the good is oft interred with their bones"; and well might a greater than Shakespeare tell his pupils to "pluck out the beams from their own eyes in order that they might see clearly to take away the motes from the eyes of their brothers."

"This is a tremendously critical age," says the superior person. That we take leave to question. It is a fault-finding age and a sceptical age if you will. But, since when did criticism begin properly to consist in mere doubting and fault-finding? The critic who can only doubt and find fault is no more than a first year's apprentice to his trade. The true and proper business, even of the critic, is to construct, or at least to point out how all things in heaven and earth ought to be constructed. Any fool can see when a tile is off the roof or the scullery window is smashed, and can tell the world with noisy and self-applauding shrieks of the wonderful discovery he has made. But is that to be called criticism? The critic who is in any sense worthy of the name can inform us why the house is ugly, and why it is uncomfortable; and can tell us, moreover, exactly how it ought to have been built in order to have been both beautiful and pleasant to live in.

The fault-finders, as distinguished from the critics, get a great deal of their own way in the world. They have two qualifications for this successful business—self-assurance and shrill tongues. If average human beings had more sense than they have, the fault-finders would soon be disposed of; the latter would find that no heed was paid to their shriekings, and that if they continued to shriek a determined, even if contemptuous, extinguisher would be put on them some fine morning. Unhappily, the quiet and duty-loving world is apt to be too modest; and, when it hears itself and its doings persistently reviled, it begins to think there may be something in the noisy accusations, and so it retires within itself more and more. That is exactly what the fault-finders want; but it is also exactly what they ought to be prevented from getting. They are the bullies of the world's school. They have learnt by experience how to do their bullying so as always to protect their own miserable skins. But a thrashing, not a silent acquiescence, is what they really ought to get as their palm of victory.

A good many fault-finding bullies gravitate towards the Press in their search for a congenial calling. The Press is a fine field for the exercise of their peculiar talents. Granted a little cleverness with a good deal of spite, and a man may find very profitable occupation in a crowded world in ministering to the numerous bullies and fault-finders of his own type. The pity is that if a newspaper or a book happens to be successful, the large public too readily concludes that it owes its success to some real worth it possesses. But a minute's reflection might show us that its prosperity may just as naturally be due to the fact that it ministers to some weakness or vice in human nature, and not at all to any high merit of its own. Newspapers and books are like businesses. A man may get rich by conducting a legitimate business; but he may also get as rich or even richer by conducting a business that is vile and mischievous. Similarly, a newspaper or a book may be influential and successful because its merits are of a great and striking order; but it may be equally or even more successful because it panders to the weaknesses, the stupidities, or the vices of its readers. All this is obvious; but, like the sun and the moon, or the wheat harvest, it is not the less important on that account.

The world is not made more cheerful by the presence of a great many fault-finders, any more than an autumn holiday is made more delightful by the presence of half a-dozen wasps' nests in the garden of the farmhouse one may have rented. It does not say much for the sober and duty-loving citizen when the fault-finder rides so prosperously in his open landau. We surely do not like to be stung with mosquitoes and worried by gnats all the day long! And yet we pay so much heed to the fault-finders of the Press that we actually make it profitable for them to continue their mean occupation.

It comes to this, as a matter of moral logic, that a man must make up his mind to do what he considers to be his duty in the world without any regard whatever for what the Press of his day may choose to say about him. If newspapers will not discriminate, then newspapers must be disregarded. In small towns and villages, where everybody is known, the slanderous tongues of gossips cannot injure the fair fame of an honourable man. Everybody knows both him and his detractors, and everybody discriminates accordingly. It is the interest of all of us to try to bring about the same state of things in larger populations. The gossiping newspaper, the paper that makes it a part of its daily business to shut its eyes to the good and to peer into every out-of-the-way corner in heaven and earth in search of the bad, must be labelled as a slanderer and a vicious shrew, and must be treated with resolute contempt by all wholesome men who stand by the worthy practice of doing as they would be done by.

For at least five thousand years moralists and philosophers have been teaching us half-a-dozen truisms of the most humiliatingly elementary kind; such, for example, as that when one man sees another man he sees a creature exceedingly like himself; or that it is a very foolish business for people who live in glass houses to make a practice of throwing stones; or that there is always the most elbowing where there is the least room; or that the man who has the keenest eye for the failings of others has had his vision sharpened by the constant necessity for watching his own. These first lines and mere rudiments of good behaviour are still unlearnt by many writers in newspapers and makers of books. It is a sorry business that of the social detective. No gentleman ever pursues it, and certainly no Christian.

